# Ethanol and biogas production after steam pretreatment of corn stover with or without the addition of sulphuric acid

**DOI:** 10.1186/1754-6834-6-11

**Published:** 2013-01-28

**Authors:** Pia-Maria Bondesson, Mats Galbe, Guido Zacchi

**Affiliations:** 1 Department of Chemical Engineering, Lund University, P.O. Box 124, SE-221 00, Lund, Sweden

**Keywords:** Corn stover, Steam pretreatment, Ethanol, Methane, Sulphuric acid

## Abstract

**Background:**

Lignocellulosic biomass, such as corn stover, is a potential raw material for ethanol production. One step in the process of producing ethanol from lignocellulose is enzymatic hydrolysis, which produces fermentable sugars from carbohydrates present in the corn stover in the form of cellulose and hemicellulose. A pretreatment step is crucial to achieve efficient conversion of lignocellulosic biomass to soluble sugars, and later ethanol. This study has investigated steam pretreatment of corn stover, with and without sulphuric acid as catalyst, and examined the effect of residence time (5–10 min) and temperature (190–210°C) on glucose and xylose recovery. The pretreatment conditions with and without dilute acid that gave the highest glucose yield were then used in subsequent experiments. Materials pretreated at the optimal conditions were subjected to simultaneous saccharification and fermentation (SSF) to produce ethanol, and remaining organic compounds were used to produce biogas by anaerobic digestion (AD).

**Results:**

The highest glucose yield achieved was 86%, obtained after pretreatment at 210°C for 10 minutes in the absence of catalyst, followed by enzymatic hydrolysis. The highest yield using sulphuric acid, 78%, was achieved using pretreatment at 200°C for 10 minutes. These two pretreatment conditions were investigated using two different process configurations. The highest ethanol and methane yields were obtained from the material pretreated in the presence of sulphuric acid. The slurry in this case was split into a solid fraction and a liquid fraction, where the solid fraction was used to produce ethanol and the liquid fraction to produce biogas. The total energy recovery in this case was 86% of the enthalpy of combustion energy in corn stover.

**Conclusions:**

The highest yield, comprising ethanol, methane and solids, was achieved using pretreatment in the presence of sulphuric acid followed by a process configuration in which the slurry from the pretreatment was divided into a solid fraction and a liquid fraction. The solid fraction was subjected to SSF, while the liquid fraction, together with the filtered residual from SSF, was used in AD. Using sulphuric acid in AD did not inhibit the reaction, which may be due to the low concentration of sulphuric acid used. In contrast, a pretreatment step without sulphuric acid resulted not only in higher concentrations of inhibitors, which affected the ethanol yield, but also in lower methane production.

## Background

Fossil-based fuels, in particular oil, dominate the transport sector. Alternatives to fossil-based fuels will become necessary as the number of vehicles increases, especially in countries with rapidly growing economies such as India and China. Furthermore, the world faces problems with greenhouse gases and diminishing oil resources. The use of biofuels, such as bioethanol and biogas, can decrease the production of greenhouse gases and reduce dependence on oil.

Ethanol can be produced from sugar, starch or lignocellulosic materials. Ethanol is currently mainly produced from sugar (sugar cane) or from starch (corn or wheat). Ethanol from lignocellulosic materials is only produced in pilot plants and demo plants. Using lignocellulosic materials, such as wood and agricultural residues, has the advantage over using sugar and starch that it minimises the conflict between using land for food production or for energy feedstock production [1]. Corn stover is a low-cost agricultural residue that is available in large quantities. Corn stover and other lignocellulosic biomasses consist of three main components: cellulose, hemicellulose and lignin. Ethanol can be produced from lignocellulose, by fermenting monomeric sugars, liberated from the cellulose and hemicellulose. Enzymatic hydrolysis is one method that can be used to convert cellulose and hemicellulose to monomeric sugars. The conversion is, however, very slow, since the cellulose is surrounded by hemicellulose and lignin, and some type of pretreatment is required [2]. Many different types of pretreatment method are used. These include pretreatment with dilute acid, steam pretreatment, wet oxidation, ammonia fibre explosion and alkaline pretreatment [2].

Steam pretreatment, also known as “steam explosion”, has been extensively investigated and tested in several pilot plants and demo plants worldwide [3]. An additional acid catalyst can be used to increase the effectiveness of the steam pretreatment, in which case hemicellulose recovery and the enzymatic hydrolysis of the solids both increase [4]. Sulphuric acid and sulphur dioxide are commonly used as acid catalysts. The pretreatment of corn stover using steam explosion with no catalyst [5,6], sulphuric acid [6,7] or sulphur dioxide [8,9] has been studied using different concentrations of the catalyst and different residence times and temperatures. However, sulphuric compounds such as sulphuric acid and sulphur dioxide must be handled in the downstream processing, and a process without sulphur is therefore preferred, if it can give similar yields and process economics as a sulphur-containing process.

The yeast strain *Saccharomyces cerevisiae* is well suited for the fermentation of pretreated and hydrolysed lignocellulosic material. Naturally occurring strains ferment glucose and mannose, but not pentoses such as xylose and arabinose. Corn stover consists of large amounts of xylose in addition to glucose, and a process that can ferment pentose sugars is essential. Several alternatives have been investigated; the use of genetically modified microorganisms to ferment pentose to ethanol [10,11], production of hydrogen [12,13] or biogas [12,14-16]. Biogas production through the anaerobic digestion (AD) of activated sludge is commonly used. The biogas can be used to produce heat or electricity, or it can be upgraded to transportation fuel [17]. Microorganisms degrade organic material to biogas during AD. Almost all organic material can be biodegraded: one exception is complicated material such as lignin [18]. Some other organic materials can be hard to degrade due to the toxic or inhibitory effects of products, resulting from previous process steps, on the organisms from, for example, phenols and some types of long-chain fatty acid [19]. Sulphide, which is produced when sulphate is reduced, can also inhibit biogas production. The main cause of inhibition is competition between sulphate-reducing bacteria and other microorganisms, in particular methane-producing organisms, for substrates. Sulphide itself is also toxic to many organisms [19]. The level of sulphides that causes inhibition has been reported to lie in the range 100–800 mg/l dissolved sulphide, and 50–400 mg/l undissociated hydrogen sulphide [19], which makes it difficult to predict the effect of pretreatment with dilute sulphuric acid or sulphur dioxide. Thus, a process that does not require sulphurous compounds is preferred, both due to the possible inhibitory effect of sulphurous compounds and due to the need to handle sulphur in the downstream processing.

The aim of the work presented here was to investigate the influence on ethanol and biogas production of steam pretreatment with or without sulphuric acid. The time, temperature and catalyst concentration during pretreatment were varied and the sugar yield determined in each case. The ethanol production by simultaneous saccharification and fermentation (SSF) and biogas production by anaerobic digestion (AD) were then studied for material that had undergone pretreatment in the conditions, both with and without acid, that gave the highest glucose yields.

## Results and discussion

### Raw material

Table 1 presents the composition of the raw material. The corn stover consisted of 34.9% glucan and starch. The amount of xylan was 18.7%. These amounts were slightly lower than other analyses of the composition of corn stover [6,8]. The amount of lignin was significantly lower than in previous analyses, due to the removal of extractives in the analytical procedure. The presence of extractives may result in too high a lignin value. 

**Table 1:** Composition of corn stover expressed as percentage of dry matter

**Glucan**	**Glucan as starch**	**Xylan**	**Arabinan**	**Galactan**	Lignin*	**Ash**	**Extractives**
33.6	1.3	18.7	2.8	1.1	14.1	2.6	17.4

*Acid-soluble lignin included.

### Pretreatment evaluation

#### Steam pretreatment

Table 2 shows the recovery of WIS and the recovery of glucan and xylan in the hydrolysate and WIS after pretreatment. The content of lignin in WIS is also shown. The maximum glucan recovery is 34.9 g per 100 g dry corn stover, recovered from both cellulose and starch. The maximum recovery of xylan is 18.7 g per 100 g dry corn stover. Table 2 shows that a recovery value above 100% was obtained in some cases, due to underestimation of the glucan content in the raw material. It is still possible, however, to compare the different pretreatment conditions.

**Table 2:** Recovery of WIS, glucan and xylan as percentage of the theoretical value, and the content of lignin in the WIS

**Catalyst**	**Temp (°C)**	**Time (min)**	**WIS (%)**	**Glucan (%)**		**Xylan (%)**		Lignin* (%)
				**WIS**	**Hydrolysate**	**WIS**	**Hydrolysate**	
None	190	5	79.8	111.0	8.9	84.6	26.8	22.6
	200	5	53.3	76.4	7.9	29.1	40.6	27.5
	210	5	62.9	106.3	7.2	21.4	36.9	28.1
	190	10	66.8	98.8	7.6	50.0	41.6	24.6
	200	10	60.6	96.5	7.6	13.0	40.3	29.2
	210	10	56.4	92.9	5.7	11.2	16.0	36.0
0.2% Sulphuric acid	190	5	70.1	97.2	7.5	32.0	60.6	27.1
	200	5	67.3	101.2	6.8	22.9	65.8	28.9
	210	5	65.9	105.2	6.3	13.8	53.1	33.0
	190	10	58.9	86.5	7.9	21.1	69.9	28.5
	200	10	65.2	104.3	6.7	14.9	42.6	27.6
	210	10	66.9	91.8	4.6	8.9	21.1	29.4
0.5% Sulphuric acid	190	5	59.9	97.6	4.2	8.5	40.2	27.2
	200	5	59.0	97.5	4.6	6.1	35.0	27.0
	210	5	62.8	106.4	4.4	4.4	25.6	28.7
	190	10	59.7	97.3	4.3	7.3	32.8	28.9
	200	10	62.9	105.1	3.9	6.2	24.1	28.1
	210	10	58.5	100.2	3.8	3.2	13.6	30.3

* Acid-soluble lignin included.

The recovery of the water-insoluble solids decreased with increasing time and temperature. The total glucan recovery was not as sensitive to harsher pretreatment conditions as the recovery of xylan.

**Figure 1:**
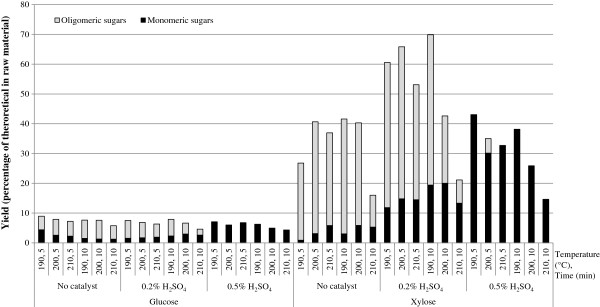
Yields of glucose and xylose in the liquid fraction of the pretreated material as percentage of the theoretical maximum from the raw material.

Figure 1 shows the amounts of xylan and glucan that were hydrolysed to oligomeric and monomeric xylose and glucose as percentages of the theoretical maximum. The amount of glucan that was found in the hydrolysate as glucose was approximately the same for all pretreatment conditions. The most notable difference is that no glucan was present as oligomers when the pretreatment used 0.5% sulphuric acid. The difference is greater for xylan in the form of xylose. Most of the glucose and xylose in the hydrolysate was present as oligomers when 0.2% or no sulphuric acid was used in the pretreatment. Harsher pretreatment conditions (higher temperature and time) initially gave higher yields of xylose, but the yield decreased when temperatures as high as 210°C for 5 minutes or 200°C for 10 minutes were reached. The xylose was degraded to sugar degradation products, such as furfural and formic acid, at these conditions. The yield of xylose in the hydrolysate was higher when 0.2% sulphuric acid was used than it was when no acid was added during pretreatment. The fraction of xylose in the form of oligomers decreased to a very small or negligible amount when a higher acid concentration (0.5%) was used. The total yield of xylose in the hydrolysate is, however, much lower than that obtained with a lower concentration of sulphuric acid.

**Figure 2:**
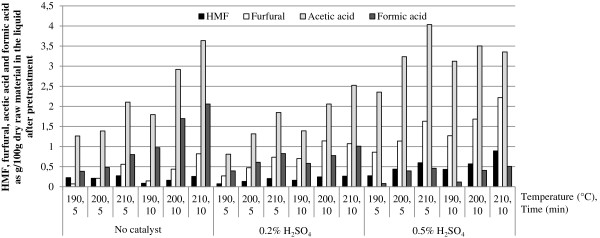
Yields of HMF, furfural, acetic acid and formic acid as g/100 g dry raw material in the liquid after pretreatment.

Figure 2 shows the concentrations of the pentose-degradation products furfural and formic acid, and the hexose-degradation product HMF in the hydrolysate as g/100 g dry corn stover. The concentration of acetic acid is also shown. Acetic acid is formed when side chains of acetyl groups are released during the solubilisation of hemicellulose. The higher concentrations of acetic acid that are produced under harsher pretreatment conditions show that more hemicellulose and, therefore, more xylan has been solubilized. The xylose yield, however, is lower, which means that more degradation products have been formed. The concentration of formic acid produced when no catalyst was used was higher than that obtained when sulphuric acid was included in the pretreatment. This shows that pretreatment without a catalyst is much harsher to hemicellulose, and degrades xylose not only to furfural, but also further to formic acid (which is a degradation product of furfural).

#### Enzymatic hydrolysis

**Figure 3:**
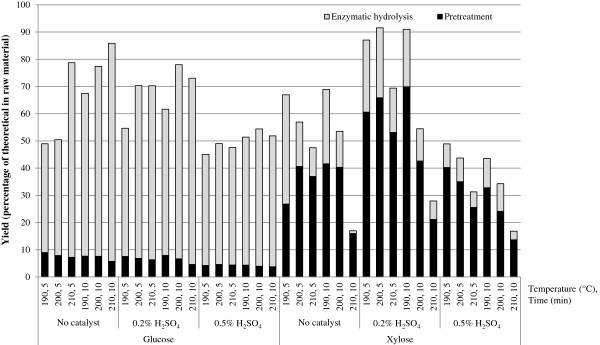
Yields of glucose and xylose in the liquid fraction after enzymatic hydrolysis of the pretreated material, as percentages of the theoretical maximum from the raw material.

Figure 3 shows the total yields of glucose and xylose after steam pretreatment and enzymatic hydrolysis as percentages of the theoretical maximum in the raw material. The highest glucose yield, 86%, was obtained for pretreatment without a catalyst at 210°C for 10 minutes. The xylose yield was lower, 17%. This is because most of the xylose has been solubilized during the pretreatment, and degraded into furfural and formic acid. A lower temperature or shorter residence time in the reactor resulted in a higher xylose yield, and a lower glucose yield. The highest glucose yield obtained when using sulphuric acid pretreatment, 78%, was using pretreatment at 200°C for 10 minutes with 0.2% sulphuric acid. The xylose yield in these conditions was 55%. This is almost the same yield as that obtained without catalyst under the same pretreatment conditions. The glucose yields are generally low, most of them being under 80%. Varga et al. [6] obtained a highest overall glucose yield of 82%, which occurred after pretreatment that included 2% sulphuric acid at 190°C for 5 minutes. The total dry matter used by Varga et al. was the same as that used here, but the enzyme loading was approximately three times higher. Varga et al. carried out enzymatic hydrolysis at 50°C, while the present study has used 40°C. Öhgren et al. [8] used corn stover pretreated with SO_2_, and showed that many different pretreatment conditions gave yields of over 80%. Their highest yield, 89%, was obtained after pretreatment at 200°C for 10 minutes using 2% SO_2_. The solid loading in the enzymatic hydrolysis was, however, only 2%, while the enzyme loading was twice as high as that used in the present study. Lloyd et al. [5] studied corn stover pretreated without the addition of acid. The glucose yield following pretreatment at 210°C for 6 minutes was lower, 67.7%, in their experiments. 

Results from enzymatic hydrolysis using unwashed material were slightly different (data not shown). The difference in yield between the highest yield for pretreatment with no catalyst and that with 0.2% sulphuric acid was smaller. Yields from unwashed material for both acid pretreatment and pretreatment without acid at 200°C for 10 minutes were also lower. There was a greater difference between the yield obtained following pretreatment with no catalyst and that obtained with 0.2% sulphuric acid, since the yield without catalyst was lower than that obtained from washed material. This may be due the inhibitor effect, since more of the hemicellulose was degraded into degradation products when no catalyst was added.

### Process evaluation

Material obtained from the pretreatment conditions that gave highest glucose yield with and without the addition of sulphuric acid was further investigated to determine its potential for producing ethanol and methane. These pretreatment conditions were 210°C for 10 minutes with no catalyst, and 200°C for 10 minutes with 0.2% sulphuric acid. The pretreatment using no catalyst at 200°C was not further investigated, since the yield was lower than that obtained at 210°C, and the total amount of inhibitors was higher than in material from pretreatment with 0.2% sulphuric acid. Table 3 presents the concentrations of sugars, degradation products and WIS in the pretreated material from the pretreatment regimens selected.

**Table 3:** Concentrations of sugars, degradation products and WIS in the pretreated material

**Catalyst**	**WIS in slurry**	**Content in WIS (% of dry weight)**			**Concentration in the liquid fraction (g/l)**					
	**(%)**	**Glucan**	**Xylan**	**Lignin**^**a**^	**Glucose**^**b**^	**Xylose**^**b**^	**Acetic acid**	**HMF**	**Furfural**	**Formic acid**
None	8.9	53.9	4.1	27.9	3.7	8.8	5.3	0.2	1.0	2.7
0.2% H_2_SO_4_	9.7	55.9	2.8	26.9	4.5	18.3	5.9	0.5	2.9	0.8

a Acid-soluble lignin included, b Monomers and oligomers.

#### SSF

**Figure 4:**
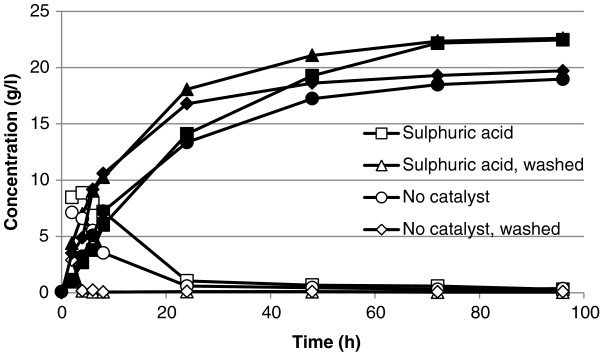
**Concentrations of ethanol (filled) and glucose (empty) in g/l for SSF performed on washed and unwashed slurry.** The shapes corresponds to the different experiment and pretreatment set up.

Figure 4 presents results from SSF. The highest concentration of ethanol, 22.6 g/l, was obtained following sulphuric acid pretreatment at 200°C for 10 minutes. The yield was the same when using washed or unwashed pretreated material. The ethanol concentration obtained was lower from pretreatment in the absence of catalyst, and in this case the ethanol concentration was lower from unwashed material than from washed material. This was due to the unwashed material containing higher concentrations of inhibitors for the yeast. The inhibitors affected also the productivity, as it took longer time to reach a given ethanol concentration. The overall ethanol yields (in percentages of theoretical maximum, based on values obtained for the glucose content in the raw material) were 80% for SSF performed on sulphuric-acid-pretreated material, 72% for material pretreated with no catalyst and subsequently washed, and 69% for the material pretreated with no catalyst and not subsequently washed. These values correspond to 16, 14.3 and 13.8 g ethanol/100 g dry raw material. The yields were similar to those obtained by Öhgren et al. from material with similar WIS content [20]. Öhgren et al. used sulphur-dioxide-pretreated corn stover, and obtained an ethanol yield of 73% using 5 g/l baker’s yeast and unwashed material. 

#### AD

Table 4 lists the VS contents and the TOC contents in the various substrates before AD. The VS content has been underestimated, since some organic acids are volatilized when the sample is dried in the oven at 105°C. The ethanol content in the substrates after SSF was measured before AD to demonstrate that most of the ethanol was distilled off during the distillation step. Table 4 presents also the VS contents of the inoculum.

**Table 4:** Contents of VS in percentage and contents of TOC and ethanol in g/l for the substrates passed to AD and in the inoculum

**Pretreatment**	**Substrate**	**VS (%)**	**TOC (g/l)**	**Ethanol (g/l)**
	Inoculum	1.36	-	-
No catalyst	Hydrolysate	1.77	10.9	-
	Thin stillage from SSF with unwashed material	3.64	14.1	0.03
	Thin stillage from SSF with washed material	1.15	5.5	0.19
0.2% Sulphuric acid	Hydrolysate	2.48	13.7	-
	Thin stillage from SSF with unwashed material	3.66	18.1	0.09
	Thin stillage from SSF with washed material	1.29	6.8	0.03

The TOC content in the inoculum-substrate mixture was measured after AD to make it possible to calculate the degree of reduction of TOC. Table 5 presents the results, together with the measured methane potentials during AD in terms of normal litre (nl) CH_4_/kg VS. The calculated potential is probably higher than the actual potential, since volatile compounds are not included in the VS measurements. The yield in terms of nl CH_4_/100 g dry raw material is, therefore, also listed, to give a more adequate comparison. All results are corrected from a blank that was run in parallel and that contained only inoculum. A reference sample containing a 50:50 mixture of two different kinds of cellulose (Microcrystalline Cellulose Powder, MP Biomedicals and Cellulose Microcrystalline, FLUKA Sigma-Aldrich Biochemika) was also run during the experiment, to ensure that the inoculum was working properly. The theoretical potential for cellulose is 415 nl CH_4_/kg VS and Table 5 shows that the result obtained was 390 nl CH_4_/kg VS. It was concluded that the inoculum being used was working properly, and any problems with the AD depended on the substrate and not on the inoculum.

**Table 5:** Contents of TOC in the mixture, degrees of TOC reduction, methane potentials and yields for the different substrates

**Pretreatment**	**Substrate**	**TOC (g/l)**	**TOC reduction (%)**	**Methane potential (nl CH**_**4**_**/kg VS)**	**Yield (nl CH**_**4**_**/100 g raw material)**
	Reference with cellulose	-	-	390	-
No catalyst	Hydrolysate	0.22	90.6	495	9.9
	Thin stillage from SSF with unwashed material	0.29	82.5	468	9.9
	Thin stillage from SSF with washed material	0.19	86.2	517	3.5
0.2% Sulphuric acid	Hydrolysate	0.02	99.0	503	11.7
	Thin stillage from SSF with unwashed material	0.17	92.1	658	13.8
	Thin stillage from SSF with washed material	0.16	91.8	516	3.8

All results have been corrected with the values obtained for the inoculum.

**Figure 5:**
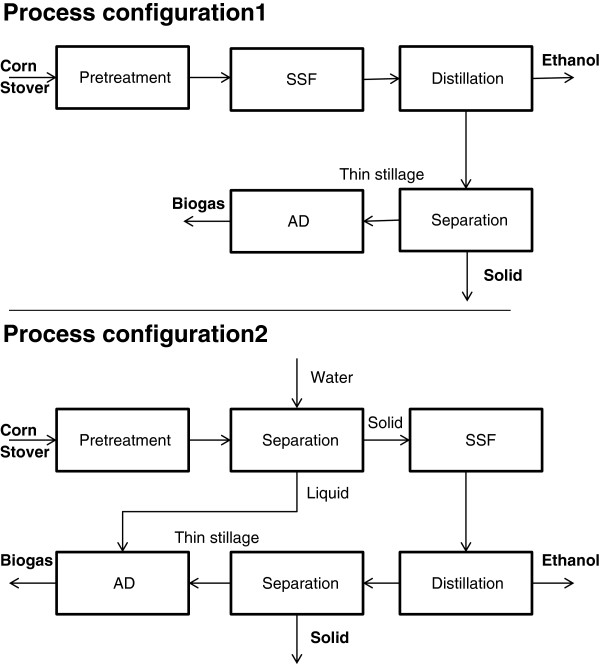
The two process configurations.

Table 5 shows that the degree of TOC reduction was high (greater than 80%) in all cases, and it was concluded that the inoculum worked well for all the substrates. The TOC reduction and the yields were higher when using sulphuric-acid-pretreated material than they were when using material pretreated in the absence of catalyst. The small amounts of sulphuric acid added during the pretreatment did not inhibit the organisms in the inoculum. Indeed – the organisms were more severely inhibited in the material pretreated with only steam, resulting in lower degrees of reduction and lower yields. This effect is compatible with problems with inhibition during SSF, and during enzymatic hydrolysis, both of which can be related to the effects of inhibitors. Two process alternatives were compared, one in which the slurry from the pretreatment was passed to SSF and from there the stillage to AD, and the other in which the hydrolysate and the thin stillage after SSF with washed material were passed to AD (Figure 5). The second alternative in which the slurry was divided into two fractions gave the highest methane yield. The result is the same regardless of whether the pretreatment was performed with or without sulphuric acid. This is probably due to the hydrolysate being diluted with washing water, which results in higher yields than those obtained with thin stillage. This would also explain the difference in the degree of TOC reduction between the hydrolysate and the thin stillage.

It is difficult to compare the results from this study with those of other studies, since most results are given as methane potential. The measurement of VS content should be corrected to account for the loss of the volatile fatty acids during the drying step to obtain better estimates of the value of VS and the following potential. This correction, however, would have no effect on the yield calculations in this study.

#### Overall product yields

**Figure 6:**
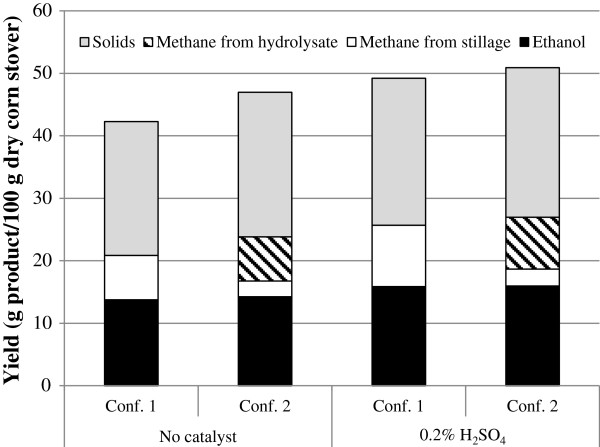
The total mass yield in g products/100 g dry raw material for the two different process configurations and the two different pretreatment conditions.

The results from SSF and AD were evaluated to compare the different pretreatment methods and process configurations. Figure 6 summarises the amounts of ethanol and methane produced, and the amounts of solids (without ash) being left for combustion. Recovery was higher from material pretreated with sulphuric acid than from material pretreated without sulphuric acid. Configuration 2, in which washed solids were used for SSF and hydrolysate for biogas, is the better option for both pretreatment methods.

**Figure 7:**
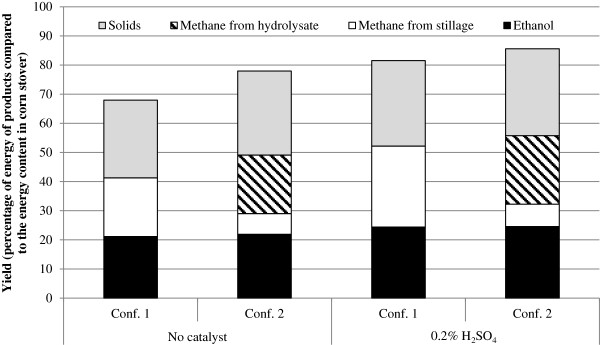
Yield in percentages of energy in products compared to the energy content of corn stover.

The combustion enthalpy in the different products was calculated and compared to the combustion enthalpy in corn stover. The energy content in the corn stover was calculated using a lower heating value (LHV) of 17.65 MJ/kg [21] and the energy contents of ethanol, methane and solid residue without ash were calculated using 27.1, 50.0 and 22.0 MJ/kg, respectively. Figure 7 shows the energy yields of the products as percentages of the energy content in corn stover. Material pretreated in the presence of sulphuric acid and subsequently undergoing Configuration 2 resulted in the highest energy yield, 86%. The energy recovery using Configuration 2 with no catalyst is better than that of Configuration 1 with sulphuric acid. 

The results show that evaluating different pretreatment conditions with enzymatic hydrolysis alone would not be a proper method. In the case of enzymatic hydrolysis, the highest glucose yield was found when no catalyst was used. This is due to the harsher pretreatment conditions that results in making cellulose more accessible to the enzymes. But when using the same conditions for SSF and AD it did not result in higher ethanol and methane yields compared with the milder pretreatment comprising sulphuric acid. The positive effect of more available cellulose for the enzymes was outweighed by the negative effects of the higher amounts of inhibitors. The amount of available residual solids that could be used for combustion was also less. Also this is due to the harsher pretreatment conditions, resulting in more lignin being solubilized. The results show that even though the optimal pretreatment conditions were chosen for highest glucose yield it might not be the optimal conditions from a process view. To find the optimum, more pretreatment conditions need to be investigated for the whole process.

Combining the production of ethanol and methane has been investigated previously. Dererie et al. [16], for example, used steam-pretreated oat straw to produce ethanol and biogas. The pretreated material was used for ethanol and the residual product for methane. The energy yield obtained by the combination was 9.5-9.8 MJ/kg dry straw, calculated from the amounts of ethanol and biogas produced. The yields from corn stover pretreated with 0.2% sulphuric acid were 9.2 and 9.8 MJ/kg dry corn stover for Configuration 1 and 2, respectively, when calculated in the same way and using same heating values as Dererie et al. Kaparaju et al. [12] obtained an energy yield of 55%, expressed as the percentage of the energy content of the dry wheat straw that was present in the products. Kaparaju et al. did not include the energy of the lignin in their calculations.

## Conclusions

Pretreatment at 210°C for 10 minutes in the absence of catalyst followed by enzymatic hydrolysis gave the highest glucose yield, 86%. The highest yield using sulphuric acid as a catalyst in the pretreatment was obtained at 200°C for 10 minutes. The highest ethanol and methane yields were obtained from material that had undergone pretreatment in the presence of sulphuric acid. The best overall yield of products was obtained from material that had undergone pretreatment in the presence of sulphuric acid, followed by processing in a configuration in which the slurry from the pretreatment was divided into a solid fraction and a liquid fraction. The solid fraction was subjected to SSF, while the liquid fraction, together with the filtered residual product after SSF, was subjected to AD. The total energy recovery in this case was 86% of the energy content in the corn stover. The use of sulphuric acid in anaerobic digestion did not give rise to an inhibitor effect, which may have been a consequence of the low concentration used. Instead, the use of steam alone in the pretreatment step resulted in a higher concentration of inhibitors, which affected not only the ethanol yield but also the methane production.

## Methods

### Process description

Corn stover was pretreated under different conditions and subsequently subjected to enzymatic hydrolysis. Material from the pretreatment conditions that gave the highest glucose yields was then input to two different process configurations, see Figure 5. In the first configuration, the slurry from the pretreatment was subjected to SSF and the filtered stillage, also known as “thin stillage”, was then passed to AD for biogas production. In the second configuration, the slurry was pressed and washed, and the solids were subjected to SSF. The hydrolysate, washing liquid and thin stillage underwent AD.

## Raw material

Corn stover was provided by the State Grid Corporation of China. The corn stover was chopped into pieces less than 20 mm and had a dry matter content of 85%. The material was stored cold. The raw material was analysed for starch, carbohydrates, lignin, ash and extractives using NREL methods [22-24].

### Steam pretreatment

Steam pretreatment was performed with only steam or with steam and sulphuric acid. When using only steam, the raw material was sprayed with water to obtain a dry matter content of about 50% by weight. When using added sulphuric acid, the raw material was impregnated with the acid. The raw material was immersed in an aqueous solution containing the concentration of 0.2% or 0.5% sulphuric acid and stored in a sealed bucket. The total weight of liquid was 20 times that of the dry corn stover. The wet corn stover was dewatered after 30 minutes in a press (Tinkturenpressen HP5M, Fischer Maschinenfabrik GMBH, Germany) of capacity 3 litres. The material was pressed to give material with a dry matter content between 45 and 50% by weight.

Steam pretreatment was performed in a reactor of capacity 10 litres loaded with impregnated corn stover corresponding to 400 g dry matter. The duration of pretreatment ranged between 5 and 10 minutes and the temperature between 190 and 210°C. Palmqvist et al. [25] describe the equipment in more detail. The solid fraction after pretreatment was analysed for structural carbohydrates and lignin, while the liquid was analysed for the contents of sugars and inhibitors using NREL methods [22,26]. The content of water-insoluble solids (WIS) was analysed using the method developed by Weiss et al. [27].

### Enzymatic hydrolysis

The pretreated material was washed with hot water before enzymatic hydrolysis. The material loading was 5% WIS. The hydrolysis experiments were performed in stirred bottles of capacity 1 litre, with a total loading of 600 g. The enzymes used, Cellic CTec2 (Novozymes, Bagsvaerd, Denmark), were added at an amount corresponding to 7.5 FPU/g WIS. Hydrolysis was allowed to continue for 96 h at 40°C. The pH was set manually to 5 with 10% sodium hydroxide. Samples were taken after 0, 2, 4, 6, 8, 24, 48, 72 and 96 h, and analysed for monomeric sugars.

### Yeast cultivation

#### Inoculum culture

Baker’s yeast (*S. cerevisiae*) produced by Jästbolaget AB, Rotebro, Sweden was prepared on an agar plate prior to the cultivation. The yeast was added to a 300 ml Erlenmeyer flask containing 70 ml sterile medium at pH 5. The composition of the medium was 23.8 g/l glucose, 10.8 g/l (NH_4_)_2_SO_4_, 5.0 g/l H_2_KPO_4_, 1.1 g/l MgSO_4_*7H_2_O. The medium also contained 14.4 ml/l trace metal solution and 1.4 ml/l vitamin solution prepared as described by Taherzadeh et al. [28]. The flask was sealed with a cotton plug and the culture was incubated at 30°C for 24 hours on a rotary shaker.

#### Aerobic batch cultivation

Aerobic batch cultivation was performed in a 2 l bioreactor (Infors AG, Bottmingen, Switzerland) at 30°C under sterile conditions. The medium contained 20.0 g/l glucose, 22.5 g/l (NH_4_)_2_SO_4_, 10.5 g/l H_2_KPO_4_, 2.2 g/l MgSO_4_*7H_2_O, 60.0 ml/l trace metal solution and 6.0 ml/l vitamin solution. The pH was maintained at 5 by the automatic addition of 10% NaOH. The cultivation was initiated by adding 60 ml of inoculum culture. The stirrer rate was maintained at 700 rpm. The bioreactor was aerated and the air flow adjusted to ensure that the concentration of dissolved oxygen was greater than 5% during the batch cultivation and the fed-batch cultivation.

#### Aerobic fed-batch cultivation

Feeding of pretreatment hydrolysate was started when the ethanol produced during the glucose consumption phase had been depleted. The hydrolysate was enriched with 62.0 g/l glucose as the concentration of hexoses was very low. Salts were added to achieve the following concentrations: 11.3 g/l (NH_4_)_2_SO_4_, 5.3 g/l H_2_KPO_4_ and 1.1 g/l MgSO_4_*7H_2_O. The total volume of the hydrolysate and salt solution was 1 l. The hydrolysate solution was added at a constant flow rate during 24 h. The hydrolysate used was diluted to a concentration that corresponded to that of a slurry from the pretreatment that had been diluted to 7.5% WIS. The pH of the hydrolysate was adjusted to 5 with 10% NaOH. The stirrer rate was maintained at 1,000 rpm.

#### Cell harvest

The cell culture was centrifuged in 750 ml flasks using a Jouan C4-12 centrifuge (St Herblain, France) at 3,500 rpm for 5 minutes. The time from the end of batch feeding to SSF of the harvested cells was never longer than 2 hours.

### Simultaneous saccharification and fermentation

Some of the pretreated material was washed before simultaneous saccharification and fermentation (SSF). The washing procedure involved first dewatering the pretreated material in a press (Tinkturenpressen HP5M, Fischer Maschinenfabrik GMBH, Germany) of capacity 3 litres to a dry matter content between 45 and 50% by weight, followed by addition of the same amount of water as had been pressed out. The material was then pressed again. SSF was performed on both washed and unwashed materials.

SSF was performed in a 2 l fermenter (Infors AG, Bottmingen, Switzerland) with a working weight of 1,000 gram. The WIS content was 8.4%, which is the highest that can be achieved when using material pretreated with only steam. This WIS was achieved by diluting the pretreated material with deionized water. The pH was adjusted to 5 with 10% NaOH, and the fermenter and the material were sterilized. The equipment was left to cool overnight. Nutrients were added to the fermenter to give concentrations of 0.5 g/l (NH_4_)_2_HPO_4_ and 0.025 g/l MgSO_4_*7H_2_O. The enzymes used, Cellic CTec2 (Novozymes, Bagsvaerd, Denmark), were added at an amount corresponding to 10 FPU/g ingoing WIS. The yeast was added to the fermenter to give a concentration of 3 g/l. SSF was performed at 35°C for 96 hours. Samples were taken after 2, 4, 6, 8, 24, 48, 72 and 96 h, and analysed by HPLC for ethanol, monomeric sugars, acetic acid, lactic acid and sugar-degradation products.

### Anaerobic digestion

The material from SSF was distilled in a small distillation unit before anaerobic digestion (AD). The distillation continued until the volume of the distillate was about 150 ml, to ensure that most of the ethanol had been removed from the slurry. The residual, the stillage, was then filtered and the liquid fraction (thin stillage) was used for AD.

AD was performed using the method described by Hansen et al. [29] to determine the potential biogas production. Either thin stillage or hydrolysate directly from the pretreatment stage was used as substrate in the AD experiments (Figure 5). The total organic carbon content and the content of volatile solids (VS) of the substrates were determined. Inoculum (active sludge) was collected from a municipal water-treatment plant (Sjölunda avloppsreningsverk, Malmö, Sweden). The VS content on the inoculum was determined. The substrate and inoculum were mixed in the proportion 1:2, measured by VS content, to give a total weight of 500 g in bottles of volume 2 l. The bottles were flushed with nitrogen to obtain an anaerobic environment, and kept in an incubator at 37°C. Samples were withdrawn twice a week and the methane content determined by gas chromatography [29].

### Analysis

Monomeric sugars from analysis of the raw material and the solids obtained from the pretreatment stage were analysed using by high-performance anion exchange chromatography coupled with pulsed amperometric detection (HPAEC-PAD). A Carbo Pac PA1 column (Dionex, Sunnyvale, CA, USA), a gradient pump (GP50, Dionex) and an autosampler (AS50, Dionex) were used. The flow rate was 1 ml/min and deionized water, 200 mmol/l sodium hydroxide and 200 mmol/l sodium hydroxide mixed with 170 mmol/l sodium acetate were used as eluents. All samples had been filtered through a filter of pore diameter 0.20 μm before analysis.

The amounts of monomeric sugars, ethanol and by products in the liquids after the pretreatment stage, after enzymatic hydrolysis and after SSF were determined by HPLC with a refractive index detector. Glucose, xylose, arabinose, galactose and mannose were separated using an Aminex HPX-87P column (Bio-Rad, Hercules, CA, USA) at 85°C with a flow rate of 0.5 ml/min using water as eluent. Ethanol, lactic acid, acetic acid, furfural and 5-hydroxymethylfurfural (HMF) were separated using an Aminex HPX-87H column (Bio-Rad, Hercules, CA, USA) at 50°C with a flow rate of 0.5 ml/min using 5 mmol/l sulphuric acid as eluent. All samples had been filtered through a filter of pore diameter 0.20 μm before analysis.

The total organic carbon content was determined by a total carbon analyser (TOC-5050A) with an autosampler (ASI-5000A). The carrier gas flow was set to 150 ml/min and the working temperature was 680°C.

The content of volatile solids, VS, was determined by ashing the sample at 550°C for 2 hours after the sample had been dried at 105°C for at least 20 hours.

## Abbreviations

AD: Anaerobic digestion; HMF: 5-hydroxymethylfurfural; HPLC: High performance liquid chromatography; LHV: Lower heating value; NREL: National Renewable Energy Laboratory; SSF: Simultaneous saccharification and fermentation; TOC: Total organic carbon; VS: Volatile solids; WIS: Water-insoluble solids.

## Competing interests

The authors declare that they have no competing interests.

## Authors’ contributions

PMB participated in the design of the study, performed the experimental work and wrote the manuscript. GZ and MG participated in the design of the study and commented on the manuscript. All authors read and approved the final manuscript.

## References

[B1] Hahn-Hagerdal B Galbe M Gorwa-Grauslund MF Liden G Zacchi G Bio-ethanol—the fuel of tomorrow from the residues of today Trends Biotechnol 2006 24 549 556 10.1016/j.tibtech.2006.10.004 17050014

[B2] Galbe M Zacchi G Pretreatment of lignocellulosic materials for efficient bioethanol production Adv Biochem Eng Biotechnol 2007 108 41 65 17646946 10.1007/10_2007_070

[B3] Galbe M Zacchi G Pretreatment: The key to efficient utilization of lignocellulosic materials Biomass Bioenergyin press

[B4] Galbe M Liden G Zacchi G Production of ethanol from biomass — Research in Sweden J Sci Ind Res 2005 64 905 919

[B5] Lloyd TA Wyman CE Combined sugar yields for dilute sulfuric acid pretreatment of corn stover followed by enzymatic hydrolysis of the remaining solids Bioresour Technol 2005 96 1967 1977 10.1016/j.biortech.2005.01.011 16112484

[B6] Varga E Reczey K Zacchi G Optimization of steam pretreatment of corn stover to enhance enzymatic digestibility Appl Biochem Biotechnol 2004 113 509 523 15054274 10.1385/abab:114:1-3:509

[B7] Tucker MP Kim KH Newman MM Nguyen QA Effects of temperature and moisture on dilute-acid steam explosion pretreatment of corn stover and cellulase enzyme digestibility Appl Biochem Biotechnol 2003 105 165 177 10.1385/ABAB:105:1-3:165 12721483

[B8] Ohgren K Galbe M Zacchi G Optimization of steam pretreatment of SO2-impregnated corn stover for fuel ethanol production Appl Biochem Biotechnol 2005 121 1055 1067 15930581 10.1385/abab:124:1-3:1055

[B9] Elander RT Dale BE Holtzapple M Ladisch MR Lee YY Mitchinson C Saddler JN Wyman CE Summary of findings from the Biomass Refining Consortium for Applied Fundamentals and Innovation (CAFI): corn stover pretreatment Cellulose 2009 16 649 659 10.1007/s10570-009-9308-y

[B10] Ohgren K Bengtsson O Gorwa-Grauslund MF Galbe M Hahn-Hagerdal B Zacchi G Simultaneous saccharification and co-fermentation of glucose and xylose in steam-pretreated corn stover at high fiber content with Saccharomyces cerevisiae TMB3400 J Biotechnol 2006 126 488 498 10.1016/j.jbiotec.2006.05.001 16828190

[B11] Olofsson K Rudolf A Lidén G Designing simultaneous saccharification and fermentation for improved xylose conversion by a recombinant strain of Saccharomyces cerevisiae J Biotechnol 2008 134 112 120 10.1016/j.jbiotec.2008.01.004 18294716

[B12] Kaparaju P Serrano M Thomsen AB Kongjan P Angelidaki I Bioethanol, biohydrogen and biogas production from wheat straw in a biorefinery concept Bioresour Technol 2009 100 2562 2568 10.1016/j.biortech.2008.11.011 19135361

[B13] Cao G Ren N Wang A Lee DJ Guo W Liu B Feng Y Zhao Q Acid hydrolysis of corn stover for biohydrogenproduction using Thermoanaerobacteriumthermosaccharolyticum W16 Int J Hydrogen Energ 2008 34 7182 7188

[B14] Bauer A Bosch P Friedl A Amon T Analysis of methane potentials of steam-exploded wheat straw and estimation of energy yields of combined ethanol and methane production J Biotechnol 2009 142 50 55 10.1016/j.jbiotec.2009.01.017 19480947

[B15] Kaparaju P Serrano M Angelidaki I Effect of reactor configuration on biogas production from wheat straw hydrolysate Bioresour Technol 2009 100 6317 6323 10.1016/j.biortech.2009.06.101 19647428

[B16] Dererie DY Trobro S Momeni MH Hansson H Blomqvist J Passoth V Schnürer A Sandgren M Ståhlberg J Improved bio-energy yields via sequential ethanol fermentation and biogas digestion of steam exploded oat straw Bioresour Technol 2011 102 4449 4455 10.1016/j.biortech.2010.12.096 21256738

[B17] Taherzadeh MJ Karimi K Pretreatment of lignocellulosic wastes to improve ethanol and biogas production: a review Int J Mol Sci 2008 9 1621 1651 10.3390/ijms9091621 19325822 PMC2635757

[B18] Frigon JC Guiot SR Biomethane production from starch and lignocellulosic crops: a comparative review Biofuels Bioproducts & Biorefining-Biofpr 2010 4 447 458 10.1002/bbb.229

[B19] Chen Y Cheng JJ Creamer KS Inhibition of anaerobic digestion process: a review Bioresour Technol 2008 99 4044 4064 10.1016/j.biortech.2007.01.057 17399981

[B20] Ohgren K Rudolf A Galbe M Zacchi G Fuel ethanol production from steam-pretreated corn stover using SSF at higher dry matter content Biomass Bioenergy 2006 30 863 869 10.1016/j.biombioe.2006.02.002

[B21] Pordesimo LO Hames BR Sokhansanj S Edens WC Variation in corn stover composition and energy content with crop maturity Biomass Bioenergy 2005 28 366 374 10.1016/j.biombioe.2004.09.003

[B22] Sluiter A Hames B Ruiz R Scarlata C Sluiter J Templeton D Crocker D Determination of Structual Carbohydrates and Lignin in Biomass 2008 Golden, CO: NREL

[B23] Ehrman T Determination of Starch in Biomass Samples by Chemical Solubilization and Enzymatic Digestion 1996 Golden, CO: NREL

[B24] Sluiter A Ruiz R Scarlata C Sluiter J Templeton D Determination of Extractives in Biomass 2005 Golden, CO: NREL

[B25] Palmqvist E Hahn-Hägerdal B Galbe M Larsson M Stenberg K Szengyel Z Tengborg C Zacchi G Design and operation of a bench-scale process development unit for the production of ethanol from lignocellulosics Bioresour Technol 1996 58 171 179 10.1016/S0960-8524(96)00096-X

[B26] Sluiter A Hames B Ruiz R Scarlata C Sluiter J Templeton D Determination of Sugars, Byproducts, and Degradation Products in Liquid Fraction Process Samples 2006 Golden, CO: NREL

[B27] Weiss ND Stickel JJ Wolfe JL Nguyen QA A simplified method for the measurement of insoluble solids in pretreated biomass slurries Appl Biochem Biotechnol 2010 162 975 987 10.1007/s12010-009-8806-6 19838648

[B28] Taherzadeh MJ Liden G Gustafsson L Niklasson C The effects of pantothenate deficiency and acetate addition on anaerobic batch fermentation of glucose by Saccharomyces cerevisiae Appl Microbiol Biotechnol 1996 46 176 182 10.1007/s002530050801 8987648

[B29] Hansen TL Schmidt JE Angelidaki I Marca E Jansen JC Mosbæk H Christensen TH Method for determination of methane potentials of solid organic waste Waste Manag 2004 24 393 400 10.1016/j.wasman.2003.09.009 15081067

